# Environmental enrichment for laboratory rats and mice: endocrine, physiological, and behavioral benefits of meeting rodents' biological needs

**DOI:** 10.3389/fvets.2025.1622417

**Published:** 2025-07-10

**Authors:** Adriana Domínguez-Oliva, Ismael Hernández-Avalos, Antonio Bueno-Nava, Cuauhtémoc Chávez, Antonio Verduzco-Mendoza, Adriana Olmos-Hernández, Dina Villanueva-García, Alberto Avila-Luna, Patricia Mora-Medina, Julio Martínez-Burnes, Arturo Gálvez-Rosas, Daniel Mota-Rojas

**Affiliations:** ^1^Programa de Doctorado en Ciencias Biológicas y de la Salud, Universidad Autónoma Metropolitana, Mexico City, Mexico; ^2^Facultad Estudios Superiores Cuautitlán, Universidad Nacional Autónoma de México (UNAM), FESC, Cuautitlán, Mexico; ^3^Division of Neurociences, Instituto Nacional de Rehabilitación-Luis Guillermo Ibarra Ibarra (INR-LGII), Mexico City, Mexico; ^4^Departamento de Ciencias Ambientales, CBS Universidad Autónoma Metropolitana-Lerma, Lerma de Villada, Mexico; ^5^Department Bioterio and Experimental Surgery, Instituto Nacional de Rehabilitación-Luis Guillermo Ibarra Ibarra (INR-LGII), Mexico City, Mexico; ^6^Division of Neonatology, Hospital Infantil de México Federico Gómez, Mexico City, Mexico; ^7^Facultad de Medicina Veterinaria y Zootecnia, Instituto de Ecología Aplicada, Universidad Autónoma de Tamaulipas, Ciudad Victoria, Mexico; ^8^Neurophysiology, Behaviour and Animal Welfare Assessment, DPAA, Universidad Autónoma Metropolitana, Mexico City, Mexico

**Keywords:** animal welfare, distress, husbandry, laboratory animals, murine, physiological traits

## Abstract

Standard housing for laboratory rodents is characterized by cages that do not always provide an adequate environment to meet the animal's behavioral needs. When animals are reared under impoverished conditions, negative states such as boredom and distress might arise. Environmental enrichment (EE) is an alternative to expose rodents to physical, sensory, cognitive, and/or social stimulation greater than the one received under standard housing conditions. The present review aims to discuss the main physiological, endocrine, and behavioral effects of environmental enrichment in murine research models. The positive and negative effects will be addressed, as well as factors including enrichment-related (i.e., type of EE, duration of EE) and animal-related aspects (i.e., strain, sex, or age) that need to be considered by researchers when adopting EE for laboratory rodents. It was observed that EE decreases corticosterone concentrations in rodents, an indication of lower levels of stress. Likewise, tachycardia, hypertension, and shorter heart rate variability are ameliorated with the implementation of EE (reflecting a beneficial effect). Among the behavioral benefits, rodents reared under EE have anti-anxiety characteristics, increased exploratory behavior, and less fear-related responses than standard-housed animals. However, in some cases, increased aggression has been reported. Although there is no standardization for EE, to properly adopt EE in experimental facilities, researchers must consider enrichment- and animal-related factors to improve the welfare of laboratory rodents.

## 1 Introduction

Currently, housing conditions for laboratory rodents have greatly improved to meet their biological needs, aiming to preserve their welfare. However, in some instances, research facilities still raise laboratory rodents in conventional or standard cages that marginally fulfill their needs by only providing absorbent bedding on the floor and an *ad libitum* food and water supply (1, 2). This type of conventional husbandry of laboratory animals is mostly based on scientific reliability to obtain high-quality, reproducibility, and optimal performance of experimental animals (3). While it provides adequate and basic physiological requirements of animals (4), this type of housing is also characterized by monotony, a sedentary lifestyle with a lack of challenges, and low cognitive and sensorial stimulation (1, 5). Moreover, captive environments prevent or decrease the presentation of the natural behavioral repertoire of the species, inducing negative mental states such as boredom, frustration, stress, and depression when highly motivated behaviors cannot be expressed (6–8). Abnormal repetitive behaviors are also associated with barren environments where animals are not interested or stimulated by their surroundings, as well as increased morbidity (5, 9). When considering the five domains of animal welfare (nutrition, environment, health, behavioral interactions, and mental state), the environment considers the animals' housing conditions (10). Therefore, impoverished housing conditions without enough positive mental stimulation are a societal and scientific concern with ethical and legal aspects that are critical to animal research (11). For example, EU Directive 2010/63 stipulates and emphasizes the need for sufficient space and environmental complexity for laboratory animals (12). Moreover, according to the principles of reducing, refinement, and replacing, improving the living conditions contributes to animal welfare and refinement in the use and care of laboratory animals (13, 14).

The proposed alternative to improve current housing conditions for laboratory rodents is environmental enrichment (EE) (6, 13, 15). The first person to acknowledge the benefits of enriching the animal's environment was Donald Hebb, who observed that pet rats reared in enriched environments from weaning (21 days) to 2.5, 15, or 25 months outperformed those housed in standard conditions. Regardless of age, pet rats had superior learning abilities, problem-solving skills, and cognitive function (16–18). According to Kentner et al. (19), 30.26% of rodents receiving EE have an age of 41–90 postnatal days (PND), followed by immediately after weaning (PND 21) to PND 40. The duration of the enrichment protocol is mostly between 4 and 6 weeks (31.43%) or 1 to 3 weeks (23.39%).

EE is a term referring to providing animals with physical, sensory, cognitive, and/or social stimulation greater than the one received under standard housing conditions (8, 9, 13, 19, 20). The Guide for the Care and Use of Laboratory Animals defines that “EE aims to enhance animal well-being by providing animals with sensory and motor stimulation, through structures and resources that facilitate the expression of species-typical behaviors and promote psychological well-being” (21). Current definitions of EE emphasize the need for enrichments to engage animals with their environment and provide information to better adapt to novelty (e.g., it must incite exploration, interaction, playing, task-solving, and learning) (7). Moreover, four different levels of EE have been described by Taylor et al. (22): (1) the so-called pseudo-enrichment (programs that do not provide any biological benefit to animals); (2) EE for meeting basic needs of laboratory animals; (3) EE aiming for hedonistic experiences such as pleasure or reward; and (4) EE aiming to have long-term accumulative outcomes on the physical and mental health of animals, including stress resilience, flexibility, and adaptability.

The principal goals of EE are to increase the behavioral diversity of the species, increase the utilization of the enclosure, prevent or decrease the presentation of abnormal behaviors, and increase the ability of the species to cope with challenges (6, 19, 23). In the case of laboratory rodents, one of the aims of EE is to encourage essential innate behaviors or species-specific behaviors such as burrowing, nest building, hiding, gnawing, grooming, digging, foraging, exploring, seeking shelter, climbing, coprophagia, and thigmotaxis (6, 19, 20, 24). Likewise, interaction with conspecifics is crucial for social species such as mice and rats (24, 25). However, these natural behaviors are limited in research facilities. In captivity, behavioral abnormalities are associated with poor environmental and cognitive stimulation (25). Common maladaptive stereotypic behavior in rodents includes wire barbering (excessive grooming), bar biting, circling, twirling, and back-flipping (5, 20). Moreover, exposure to chronic stressful environments (e.g., impoverished cages that do not meet animals' biological needs) predisposes them to neurochemical, endocrine, physiological, immune, and behavioral alterations (26).

Natural behavioral repertoires can be motivated through EE strategies that combine physical exercise, interaction with conspecifics or with humans (e.g., positive reinforcement training), larger spaces, and complex and constantly changing novel stimuli (24, 27, 28). EE can be divided into physical and social enrichment, although the stimuli provided can be subdivided into sensory, structural, social, occupational, feeding-based, or a combination of all (7). Physical enrichment refers to structural modifications mostly observed as larger cages or increased floor space. Moreover, the addition of objects to encourage exercise, play, and exploration is another type of physical enrichment for laboratory rodents (19, 25). Among these objects, shelters, dens, hideouts, nest boxes, and nesting material (paper, fiber-based) are some of the main enrichments due to their importance as behavioral needs of the species (rodents spend almost 20% of their time budget interacting with nesting materials) (24, 25, 29). Rodents have a strong motivation to nest even among non-breeding animals (15).

The addition of toys such as wooden objects, balls, bones/chews, or chewing materials responds to the need of rodents to gnaw on their continuously growing incisors (2, 19, 27). Rodents also need burrowing substrates and foraging opportunities provided as scattered food or natural treats (sunflower seeds, oat flakes, hazelnuts, cashews, and almonds) (2, 30). Providing a greater variety of foods to rats stimulates foraging and, in consequence, physical condition (31).

Likewise, platforms, ropes, swings, running wheels, and ladders actively motivate the animals to exercise and stimulate playful behavior (19, 24, 25, 29, 32). These items can be provided permanently inside the animal's cage or rotated to avoid habituation to the object (33). Rodents quickly adapt to novel environments, and the effect of EE might cease over time. Thus, novelty and cage-changing routines might help to maintain the positive effects of EE (34). In the case of plastic tunnels or shelters, rodents as prey species seek hiding places to flee and hide from predators (15). Thus, the physical enrichment provided to rats and mice in research centers must replicate the natural behaviors of the species (31). On the other hand, social enrichment refers to housing social animals in groups wherever possible or by interacting with humans (20, 33). For example, 1% of studies focusing on EE group-house the animals (2). Table 1 summarizes the main items used for EE of laboratory rodents.

**Table 1:** Main items used for environmental enrichment of laboratory rodents according to their purpose (9, 19, 34).

**Purpose of the enrichment**	**Recommended items**
Additional space	Larger cages
	Different levels
	Segregate areas
	Ladders
Hiding places	Tubes
	Pipes
	Tunnels
	Cardboard houses
	Plastic houses
	Shelters
Nesting materials	Nest packs
	Bedding
	Nestlets
	Cocoons
Gnawing objects	Wooden blocks
	Chews
Foraging opportunities	Scattering food
	Treats
Toys	Running wheels
	Crawling balls
Climbing	Ropes
	Chains
	Wood scaffold
	Hemp rope
Auditory stimulation	Bell
	Chimes
	Music
	Sounds
Olfactory stimulation	Scents
	Aromatherapy
	Essential oils
	Lavender
Tactile stimulation	Handling
	Brushing
Cognitive stimulation	Solving tasks
	Puzzles
Social interaction	Housing more than one animal per cage
	Reinforcement training

When the environment of laboratory rodents is enriched, several neurobiological, physical, immune, and behavioral benefits have been reported (26, 33). Among these, increased behavioral diversity and the animals' coping ability when facing challenges or distress (1, 6, 31). The physiological response of animals to challenges also improves when exposed to EE. Regarding behavioral outcomes, physical and psychological health improve, reducing the presentation of abnormal repetitive behaviors (6, 13, 31). Depressive and anxiety-like behaviors also diminish (32).

Adopting EE protocols has gained importance in the last decade. In 2022, 122 publications regarding environmental enrichment were published (35). However, although there is a large body of evidence showing the benefits of EE, certain challenges remain under research, such as effects of age, sex, strain, and species (5). For example, in male mice housed in cages with physical and social enrichment, the addition of EE causes negative effects by inducing agonistic encounters, increasing anxiety-like behaviors, and higher corticosterone (CORT) levels (32, 36). Thus, as Würbel and Novak (9) mention, an adequate EE protocol must not only encourage natural behaviors but also reduce abnormal behaviors. Moreover, one of the reasons why researchers are reluctant to adopt EE is due to the variation that non-standard housing might pose to the experimental results. Nonetheless, it has been shown that the beneficial effects of EE can be met without reducing the reproducibility and validity of the results (37). The present review aims to discuss the main physiological, endocrine, and behavioral effects of environmental enrichment in murine research models. The positive and negative effects will be addressed, as well as factors including enrichment-related (i.e., type of EE, duration of EE) and animal-related aspects (i.e., strain, sex, or age) that need to be considered by researchers when adopting EE for laboratory rodents.

## 2 Search methodology of the present review

A literature search was performed using Web of Science, PubMed, MEDLINE, and PsycINFO. The following keywords were used in combination to find the papers: “environmental enrichment” “laboratory rodents”, “rats”, “mice”, “corticosterone”, “stress-related hormones”, “physiological changes”, “heart rate”, “heart rate variability”, “stereotypies”, “abnormal behavior”, and “anxiety-like behavior”. There was no limited publishing date to cover relevant papers regarding EE. Selected studies were those written in English and with full text available. The selected papers were those in which EE was provided to clinically healthy rats and mice. Studies where animals were used as a disease model of interest (e.g., traumatic brain injury, autism, diabetes, among others) or received any kind of drug were excluded, as the aim of the current paper was to focus on the effect of EE on all rodents and not only under certain pathological conditions.

## 3 Stress-related systems and environmental enrichment for rats and mice

**Figure 1:**
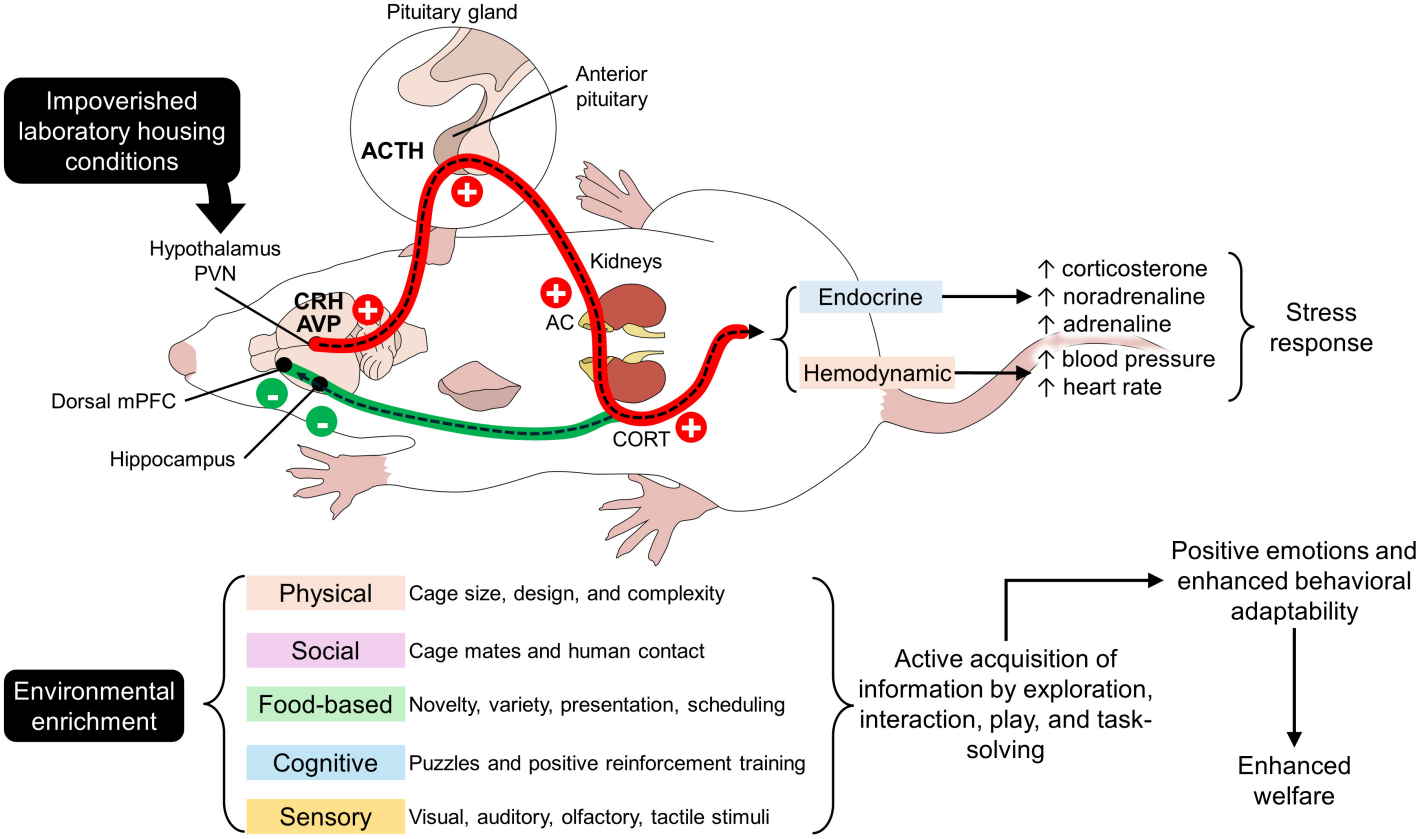
Hypothalamic-pituitary-adrenal axis (HPA) and environmental enrichment. Impoverished housing conditions in laboratories activate the main axes related to stress. Stressors stimulate the neurons in the paraventricular nucleus (PVN) of the hypothalamus to secrete corticotropin-releasing hormone (CRH). These neurons project to the anterior pituitary to release adrenocorticotropic hormone (ACTH) directly into the adrenal cortex (AC). Activation of the AC culminates in the secretion of glucocorticoids (corticosterone, CORT), the main biomarker of stress in rodents. This cascade causes a series of endocrine and hemodynamic changes that modify the animals' physiology and behavior (this pathway is marked by the red line). In contrast, when environmental enrichment is provided through physical, social, food-based, cognitive, and sensory stimuli, the HPA axis goes into a negative feedback loop. CORT inhibits glucocorticoid receptors in the hippocampus and the prefrontal cortex (mPFC) when the environment does not elicit a stress response (this pathway is indicated in green). Plus and minus signs indicate the positive and negative activation of the HPA axis.

Stress is the non-specific response of the organism to physical or psychological disturbances and environmental demands (38). Stress is manifested through physiological and behavioral responses that can be positive (eustress) or negative (distress) (25). Eustress refers to a functional and constructive type of stress that promotes growth and learning. In contrast, distress is the response to actual threatening situations that hinders the overall performance of animals (39, 40). It has been previously mentioned that impoverished standard cages might cause frustration, depression, boredom, and distress in animals that cannot meet their behavioral needs (6–8). As shown in Figure 1, the perception of a stressor—barren cages for laboratory rodents—can directly activate the stress systems in mammals, mainly the hypothalamic-pituitary-adrenal (HPA) axis and the corticotropin-releasing hormone and the adrenocorticotropic hormone (ACTH) (41). This causes the secretion of neuroendocrine mediators such as CORT in rodents (38), among increases in other mediators such as catecholamines and decreases in oxytocin and testosterone levels (38). Environmental stressors also increase the activity of the sympathetic nervous system, affecting cardiovascular parameters such as blood pressure (BP, increase), heart rate (HR, increase), and heart rate variability (HRV, decrease) (42), which will be discussed in the following lines.

The main neuroendocrine marker for stress in rodents is CORT. It can be measured in blood plasma (32, 43), as well as fecal/urine metabolites (38, 44) and hair samples (3, 30). Each method of CORT measurement is used to evaluate the acute stress response of rodents immediately after a stressor (blood plasma), after a couple of hours (feces/urine), and for chronic exposure to stressors (hair) (32, 43, 45, 46). EE is frequently associated with improved stress resilience in animals, thus decreasing the levels of CORT (32). This was reported in male Long Evans and Sprague-Dawley aged 4–6 months. The animals were exposed to social (housed in groups of four to five individuals per cage) and structural enrichment (grids for climbing, metal running wheels, climbing ropes, cloth hammocks, plastic tubes, and toys), followed by a restraint test to compare the stress-related response. Blood plasma CORT levels were higher in control groups (non-enriched groups) at 30 and 60 min after restraint (~400 vs. 300 ng/ml, respectively). Moreover, CORT values in the EE groups returned to baseline levels more quickly than in the control group (25).

In another study, male Long-Evans rats of 35 to 38 PND were housed under physical (e.g., ladders, climbing tubes, cotton balls, running balls, plastic hideaways) and social enrichment (pair-housed) for 6 weeks. To assess the effect of acute and chronic stress (predator sounds for 30 min/daily), the authors compared the levels of fecal steroid hormones (CORT, dehydroepiandrosterone (DHEA), and testosterone) in animals with and without EE. The rats exposed to chronic and acute stress with EE had lower CORT values than those without EE (up to 1,800 vs. 2,700 μm/mg, respectively). Additionally, DHEA levels increased significantly for animals exposed to acute and chronic stress without EE (38). These results are significant because CORT and other metabolites, such as high DHEA levels, are considered biomarkers for stress (47).

In male CD-1 mice, McQuaid et al. (32) reported that social (three mice/cage) and structural enrichment (large cage, running wheels, shelters, tunnels, two cotton nestlets) for 6 weeks minimized the stress response of mice aged 21 PND. Basal levels of blood plasma CORT in enriched animals were lower than those in the control group (housed in standard cages with one cotton nestlet) (6 vs. 7 ug/dl, respectively). When facing a social stressor (measured through the social interaction test), the EE group had significantly lower values than control animals (~6 vs. 12 ug/dl, respectively). Similar results were obtained by Meijer et al. (48) in female mice of the C57BL6/Jico inbred strain. Mice were individually housed for 3 weeks with physical enrichments (paper-based house, Kleenex^®^ tissues, EnviroDry^®^, PVC tube, and chew aspen sticks). Although no significant changes were recorded, the blood CORT of enriched animals tended to have lower values than those maintained in unenriched conditions with only aspen chips bedding (40 vs. up to 115 nmol/l, respectively). Likewise, in 35-day-old female BALB/c and C57BL/6 strain mice, the effect of physical EE (shelters, tubes, paper balls, and nesting material) on blood pressure and CORT blood serum was evaluated for 6 weeks. EE significantly reduced the blood plasma levels of CORT (0.6 vs. up to 1.2 μg/dl, respectively) and blood pressure (~100 vs. 130 mmHg, respectively) compared to control animals maintained in standard laboratory cages (43).

Although previous studies have shown that CORT levels decrease in animals receiving EE, this response might be sex-dependent, as some studies comparing females and males have reported controversial results. An example is Elmi et al. (3), who compared hair CORT and DHEA values of outbred male and female Wistar rats. Cages with 28 PND Wistar rats housed in groups were equipped with tunnels (physical EE) for 3 months. The authors used two types of EE: a group with only one tunnel and another one with an additional tunnel hanging from the cage. While no differences between enrichments were found, females had higher levels of CORT and DHE than males (CORT between 365.89 ± 95.95 and 380.40 ± 53.39 pg/ng; DHE between 31.33 ± 7.67 and 52.46 ± 15.37 pg/mg). In contrast, males of the group with two tunnels had higher CORT levels than EE animals (380.40 ± 53.39 vs. 365.89 ± 95.95 pg/mg, respectively). Similarly, adolescent (23 PND) male and female Wistar rats were housed in enriched or control conditions (standard laboratory cages) for 10 weeks. The EE included physical (tunnels, non-chewable toys, climbing platforms, and running wheels) and social enrichment (five rats per cage). On postnatal day 107, animals were exposed to an acute stressor (10-min period of immersion in water) to assess the neuroendocrine response. Serum CORT values of males were significantly lower (695.92 ± 27.03 ng/ml) than females (1030.65 ± 27.05 ng/ml). Moreover, females receiving chronic stress in control cages had lower values than those exposed to EE (1007.71 ± 14.33 vs.1160.71 ± 18.68 ng/ml, respectively) (49). The differences according to sex might be related to the different stress thresholds between males and females (considering that novel EE items might be perceived as a stressor) (50, 51). However, the time of sampling collection might also be associated with the sex differences. In this sense, in the previous study, CORT evaluation was performed at PND 107, while EE started at PND 24, which might indicate that females were rather more stressed than males after the addition of EE in general, without involving the novelty aspect. Additionally, advanced studies performed by Lin et al. (50) have found that physical and social EE (five mice per cage, housed in plastic tubs with running wheels, habitrails, and plastic objects) reduces anxiety in males but is anxiogenic in female C57BL/6J mice due to increased hippocampal steroid receptor ratio in females. Additionally, the same authors mentioned that social enrichment or group-housing possibly positively influences females more than males.

Studies have also reported no differences in CORT levels when comparing rodents housed in control conditions and those under EE protocols, as shown by Schrijver et al. (52) in male Lister hooded rats of 21 PND. Rats were individually or group housed in control (Makrolon type IV cages with sawdust as bedding) and enriched cages until week 12, when a restraint test was used to assess the HPA activity. Enriched condition was housing rats in rabbit cages with a thick layer of bedding material, shelves, hay, rope, plastic tunnels, and huts. Although significant and general increases between basal levels of blood plasma ACTH and CORT were observed during the restraint stress (from 119.12 ± 3.34 to 375.74 ± 33.05 pg/ml and from 71.06 ± 5.55 to 207.19 ± 5.80 ng/ml, respectively), the levels of blood plasma ACTH and CORT were similar between the control groups (up to between 350–500 pg/ml and 200 ng/ml, respectively) and enriched groups (approximately 350 pg/ml and 200 ng/ml, respectively). Additionally, rats exposed to EE had an attenuated ACTH peak response compared to control cages. Similarly, Roschke et al. (30) evaluated the effect of EE in male Sprague Dawley rats aged 6–8 months through measurements of hair testosterone, DHEA, and CORT. Social and physical enrichment (with enriched houses with additional floors, and access to a playpen) was provided. No statistically significant differences were observed with control animals reared in standard Makrolon type IV cages with bedding. However, DHEA levels were significantly higher in the enriched group with access to a playpen (above 3.25 pg/mg), while testosterone levels decreased in the control group (1.08 pg/mg), both biomarkers of stress.

In addition, Muthmainah et al. (53) compared three experimental groups of male Wistar rats of 42 PND: a control group (standard cage), rats exposed to chronic stress (unpredictable mild stress protocol), and rats exposed to chronic stress but with EE. Social and physical enrichment (larger cages with small balls, slides, running wheels, plastic tubes, bedding material, and ladders) was applied for 21 days. The authors did not find significant differences in blood plasma CORT concentrations among groups, results that are similar to those reported by Kelogan-Musuroglu et al. (54) in Wistar albino male rats under social isolation with and without EE. In the case of male mice of inbred strain ABG of 21 days of age, when comparing control conditions (Makrolon type II cages with wood shavings) vs. enriched (box and scaffolding) and super-enriched housing (spacious glass terrariums with extra plains, wooden footpaths, hemp ropes, climbing tree) until PND 77± 3, no differences in blood plasma CORT were found (55). The lack of significant differences might be due to the influence that EE has on social relationships, as mentioned by van de Weerd et al. (56) when providing nesting material (Kleenex^®^ tissues) to socially housed male and female C57BL/6NCrlBR and BALB/cAnNCrlBR mice. In these animals, no differences in blood plasma and urine CORT were observed when compared to control housing (Makrolon type II cage with sawdust bedding).

In some other instances, CORT increases in animals reared under enriched conditions. This was observed in 80-day-old CD-1 male rats who received EE for 33 days. Enriched conditions consisted of larger cages of two levels and one PVC tube. Control rats were allocated to standard cages of one level with one PVC tube. Fecal CORT values were higher in the enriched group (~120 ng/g) than in control animals (~60 ng/g) (44). Likewise, Mesa-Gresa et al. (26) compared a group of chronic social stress (housing four animals from different cages) in NMRI male mice at 28 days of life and an EE group. EE included larger cages with houses, tunnels, running wheels, and toys. In contrast, control housing was characterized by standard cages with only sawdust. After providing EE until 77 PND, enriched-housed mice had higher CORT levels (224.13 ± 21.22 ng/ml) than those allocated to control conditions (172.71 ± 13.99 ng/ml). Increases in urine CORT were also reported by Hutchinson et al. (57), who mention that providing enrichment to female mice might cause endocrine alterations. The authors compared no enrichment (control group housed in standard cages with only aspen bedding chips), nesting material (animals receiving only a compressed cotton nesting pad), and ‘super enrichment' (aspen bedding, nesting pad, plastic hut, and two plastic balls) in female BALB/c mice of 21 PND. Physical and social enrichment (five mice per cage) was provided until week 14. The authors evaluated urine CORT and reported that mice receiving nesting material had higher urinary CORT (99.8 ± 4.0 mg/mol) than control mice (83.6 ± 4.0 mg/mol).

The contradictory endocrine response observed in some cases might be due to the role of CORT as a hormone that indicates arousal, whether negative or positive (e.g., playing, exercise, sexual interaction) (44). Moreover, the addition of EE might also increase agonistic interactions between animals fighting for the added item (55) or might be due to preferences according to the species. For example, some nesting material can be unsuitable for some animals (e.g., cotton nesting pads are recommended for rodents), which might cause distress (57). Additionally, overly complex environments provided through EE might be perceived as a stressor when the available space seems to be reduced, which might induce chronic stress and have a negative impact on animals. However, further research is needed to elucidate the association between glucocorticoids and housing conditions in laboratory rodents. Therefore, although CORT is a valuable indicator of the activation of the HPA axis, the evaluation of the EE must be performed together with other variables (e.g., physiological parameters and behavioral responses) to determine the positive, neutral, or negative effect of EE on laboratory rodents.

## 4 Physiological benefits of environmental enrichment

In addition to the CORT levels, the physiological response of rodents to different environmental factors (including EE) has been studied—although not as extensively as the endocrine and behavioral response (42). Stress can influence the cardiovascular function by increasing the activity of the autonomic nervous system (ANS) and the release of catecholamines (noradrenaline and adrenaline) by the sympathetic branch (SNS). These responses are characterized by hypertension and tachycardia (58). In the case of female mice of the C57BL6/Jico inbred strain at 21 days old, physical and social enrichment (three animals per cage) was provided up to week 15. EE consisted of Makrolon type II cages with aspen bedding, paper houses, tissues, PVC tubes, and chew sticks, while control animals were housed in Makrolon type II cages with aspen chips bedding. The authors evaluated the effect of housing on animals' HR. The animals of the enrichment group had a lower mean baseline HR (500–550 bpm) than the animals maintained under control conditions (around 600 bpm). Moreover, after the restraint test, body temperature of the enriched group (obtained through an implanted peritoneal transmitter) was significantly higher than the standard group (up to 37.7 vs. 36.4°C, respectively) (48). Similarly, 12-week-old male mice (NMRI) housed individually had higher HR than those housed in pairs with a female (501 to 618 vs. 488 to 594 bpm, respectively). In addition, body temperature was lower in individually housed animals (35.7–36.9°C) than in mice housed in pairs (35.7–37.9°C) (59). These studies show that basic physiological parameters in laboratory animals have housing-dependent variations.

Heart rate variability (HRV) is a metric that has been used to assess the physiological response of animals to EE because it changes according to the activation of the ANS (60). HRV is a measurement that reflects the activity of the sympathetic versus the parasympathetic nervous system (42). Following the cardiovascular response of animals under distress, tachycardia decreases the HRV (61). Studies performed in rats exposed to acute and chronic stress (food and water deprivation, wet and tilted cage, forced swimming, and restraint) found that long-term stress causes a dysfunction in the parasympathetic function and hyperactivation of the SNS (62). Brauner et al. (42) evaluated the changes in HRV in male Sprague-Dawley rats (1–2 years old) during 3 weeks. The authors used physical (tubes and shelves) and social enrichment (pair-housing) and compared the HRV values of enriched vs. control rats (reared in small cages with pine shavings as bedding). The results showed that EE significantly reduced the sympathetic activity (reduced the low frequency/high frequency ratio (LF/HF), parasympathetic predominance) while control animals had a high LF/HF ratio (sympathetic predominance). Similarly, social enrichment was provided for 10 days to female prairie voles (60–90 days old) (63). Socially enriched animals had increased HRV and decreased HR when compared to isolated animals. As mentioned by Kapusta et al. (64), one of the main goals of EE is to reduce animals' stress, which can be reflected as a lower sympathetic activity.

Another crucial aspect of EE is the difference in body weight (BW) between rodents receiving EE and those in standard housing. This is relevant because some types of enrichments are food-based and might predispose to significant weight gain, as found in 4–6-month-old Long Evans and Sprague-Dawley rats exposed to 6 weeks of social and physical EE (25). Group-housed animals (four or five per cage) were allocated in large cages with metal running wheels, climbing ropes, cloth hammocks, and plastic tubes. BW increased by 18% and 12%, respectively, while non-enriched groups (singly housed in polycarbonate cages) only recorded increases of 10%. An increase in BW was also reported in another study made in C57BL/6J female mice after 29 weeks of EE using running wheels, mouse igloo, paper nesting, and cotton rolls in addition to other materials. Enriched housed mice had significantly higher BW (24.99 g) than animals housed in control conditions with aspen bedding material, paper and cotton nesting material (23.66 g) (13). Similarly, higher BW were recorded in adolescent (23 PND) male and female Wistar rats housed in enriched large cages with tunnels, non-chewable toys, climbing platforms, and running wheels, than in those reared under control housing conditions for 10 weeks. After exposing the animals to a 4-week chronic stress protocol on PND 66 (i.e., restraint stress, cage rotation, social isolation, among others), rats receiving EE without stress had the highest BW (30%), followed by those not receiving EE and those receiving EE and exposed to chronic stress (25%) (49).

On the contrary, some authors refer that enriched conditions increase animal activity in enlarged cages and might significantly reduce the BW of the experimental animals. In this sense, lower BW was reported by Mesa-Gresa et al. (26), who compared NMRI male mice of 28 PND housed in chronic social stress (house changing with unfamiliar mice) or under EE conditions (large cages with houses, tunnels, running wheels, and toys). After 49 days of EE, enriched animals gain less weight than control animals (standard cages with only sawdust). In juvenile male Sprague–Dawley rats, Zaias et al. (65) provided social (multiple housing) and physical enrichment (igloos, tunnels, nesting material) from PND 23 to 45. Within 24 h of housing (PND 24), the EE group weighed an average of 4 g less than the control group housed in a standard polycarbonate shoebox with aspen chip bedding. These differences might be related to the larger available space and increased physical activity of animals, as shown when comparing the provision of only one physical enrichment vs. multiple items to newly weaned Wistar male rats for 5 weeks (37). In this study, multiple items (e.g., ladder, crawling balls, and wooden blocks) reduced feeding time (9.22 ± 0.88 vs. up to 15.63 ± 1.34%, respectively), which indicated that animals spend less time feeding and more time interacting with the enriched environment (37).

Additionally, other studies have found no significant differences in BW between enriched- and control animals. For example, Meijer et al. (48) evaluated the effect of EE on the stress response (short periods of restraint) of female mice of the C57BL6/Jico inbred strain (3-week-old). After physical EE was provided up to week 15 (cages with aspen chips bedding, paper houses, tissues, and PVC tunnels), BW was similar between the enriched and the control group (Makrolon type II cages with aspen bedding). Likewise, male NSY/Hos mice (98-day-old) did not show BW differences when evaluating the addition of nest boxes to individually-housed mice (physical enrichment) (66). After 5 weeks of EE, the control group weighed between 40.9 ± 2.0 and 43.1 ± 3.1 g, while the enriched group recorded weights between 41.6 ± 2.2 and 43.4 ± 4.3 g with no significant differences.

## 5 Behavioral benefits of environmental enrichment

One of the main goals of EE is to encourage behavioral diversity and increase animals' normal (species-specific) behavioral repertoire (37). For example, Rojas-Carvajal et al. (27) found that physical activity and cage exploration (i.e., sniffing) were the most frequent behaviors in male Wistar rats of 29 days old enriched in large wire-mesh cages containing dens, hideouts, nesting, and chewing materials. In the same species, Abou-Ismail (37) reported the effect of adding one item (ladders, crawling balls, nylabones, wooden blocks) or multiple items per cage for 5 weeks to newly weaned Wistar male rats (PND 28). In rats enriched with multiple items, behaviors such as sleep (45.78 ± 2.16%) and grooming (21.13 ± 1.33%) increased. In contrast, agonistic encounters significantly decreased in rats enriched with several objects than those provided by only one enrichment (2.50 ± 0.45 vs. up to 10.73 ± 0.89%, respectively). In this study, the increase in grooming behavior might be related to overall welfare, as self-grooming is considered an innate behavior linked to comfort due to its role in thermoregulation, hygiene, and emotional regulation (67, 68). Likewise, the high level of sleep displayed by rats interacting with multiple items might be related to the increased activity level or low anxiety levels (37). Play behavior (hopping) and general activity also increased in male mice of the inbred strain ABG exposed to social (groups of four animals per cage) and physical EE (plastic inset, wooden scaffolding, plastic stairs, hemp ropes, climbing tree) from weaning (22 ± 1 days) until PND 77 ± 3 days (+50%) (55).

A reduction in agonistic behavior was also observed in newly weaned male Wistar rats provided with physical EE, including a complex cage, aspen wooden blocks, shelters, polycarbonate balls, and ladders (8). The authors found that cage compartmentalization serves as a refuge from conspecifics, thus decreasing agonistic interactions. Similarly, social dominance is highly influenced by EE. Male Wistar rats of 21–23 PND were exposed to different housing systems (individual vs. social housing, with or without enrichment) from weaning (21–23 days) to 49 PND (69). Group-housed animals (six rats per cage) were enriched with wooden materials, plastic objects, swings, climbing frames, and elevated shelters (physical enrichment). When forcing an encounter between two unfamiliar animals, EE suppressed dominant behavior in individually housed rats (30%) when compared to control housing (68% in animals reared in standard cages). However, social housing combined with physical EE was more effective in reducing dominant behavior (45%) when compared to individually-housed rats with EE (52%) (69). Moreover, similar to what was found in rats, social housing (five animals per cage) and physical EE (Compressed nesting pads, plastic huts, and plastic balls) in female BALB/c mice of 21 PND (provided until week 14) reduced tail-wound scores in enriched animals (1.27 ± 0.08–1.2 ± 0.09) when compared to non-enriched mice (standard cages with aspen bedding chips, 1.68 ± 0.12) (57). These results show the importance of socially housing animals whenever possible, as social EE has a greater effect on rodents, a species that lives in groups with complex structures and interactions (31, 70, 71).

When assessing the effect of EE, the animal's natural repertoire and preferences must be considered to design an appropriate enriched cage. This was reported by Roschke et al. (30) in male Sprague Dawley rats aged 6–8 months receiving social and physical enrichment (cages with additional floors and access to a playpen). It was observed that rats extensively used hiding houses and tubes while vertical climbing was rarely used. Regarding the rodent treats, almonds and hazelnuts were preferred over cashew nuts. Preference was also investigated by Kawakami et al. (72) in male C57BL/6J mice, where it was found that these animals (known to have dark fur) preferred to stay in dark cloth when provided with three different bedding materials (wood shavings, paper, and cloth). C57BL/6J mice stayed longer times in the dark cloth (up to 600 min/720 min). In contrast, ICR male mice (species with white fur) preferred the white cloth, staying there for approximately up to 500 min/720 min. The authors concluded that selection and preference for certain enrichment is related to the fur color of the animal by camouflaging with the environment and preventing predation (73). Additionally, in female C57BL/6J and DBA/2N mice, Bohn et al. (34) found that mice prefer EE items (e.g., paper towel, wooden gnawing sticks, plastic houses, and tunnels) over additional space when assessing spontaneous behaviors of animals maintained in double cages with access to an extra cage (with or without items). The results showed that mice preferred the extra cages with EE items (up to 60% of their time) over cages without EE.

Another of the main goals of EE is to reduce the presentation of stereotypies (64). Stereotypies are repetitive behaviors or invariant motor patterns that lack an apparent function (6, 74). Examples include jumping or bar-mouthing in mice (74). They are often observed in laboratory rodents when housed in impoverished environments with limited space and a lack of complexity (31). Although several factors are associated with the presentation of stereotypies, they reflect compromised welfare in laboratory rodents (75). Several studies have reported the beneficial effect that EE regarding stereotypies, as Meira et al. (31) found after providing physical (plastic tubes) and feeding (cardboard roll filled with sunflower seeds) EE to male Wistar rats of 200 g. At the end of the experimental phase (10 days), rats reared under EE protocols had lower frequencies of gnawing the grids (0–0.17) than animals reared in control conditions with standard cages and wood shavings (0.33). Moreover, stereotypic movements were completely absent in enriched animals, while control animals had a frequency of 2.50.

Hobbiesiefken et al. (13) evaluated 18 weeks of physical EE (nesting material, tunnels, and plastic houses) in C57BL/67 female mice. Stereotypical behaviors (i.e., scratching, wiping, bar-orientated behavior, circling, jumping, and route tracing) significantly decreased when compared to control housing animals (groups of four in Makrolon type III cages with aspen bedding) (4 vs. 36%, respectively), while feeding behavior increased up to 20%. Similarly, female ICR CD-1 mice of 21 days old were maintained in group-housed enriched enclosures with shelters, climbing structures, and nesting materials to measure the stereotypy level (74). At 6 months old, control mice spent 22.67% of their time performing stereotypies, while EE animals recorded only 13.58 ± 5.2%. Similarly, at 11 months, control mice spent more time presenting stereotypies than EE mice (20.78 ± 5 vs. 12.09 ± 4.1%, respectively). Stereotypies included bar mouthing, circling, backflipping, patterned running, and patterned climbing (74).

The effect of EE on stereotypic wire-gnawing behavior was studied by Würbel et al. (76) by comparing enriched 21-day-old male mice of the ICR strain (cardboard tube) with control animals (barren standard cages). The results showed that enrichment significantly reduced the presentation of stereotypies by 40%. Similarly, in male BALB/c mice (21 PND), Leach et al. (23) compared an enriched environment with wood blocks and a plastic insert with control conditions (cage with paper bedding) to determine its effect on different behavioral responses, including bar gnawing. Enriched animals significantly reduced the frequency of bar gnawing (0.87) when compared to control animals (7.62). Moreover, animals with EE had higher frequencies for exploration (2.84). Motor stereotypical jumping and somersaulting were also reduced in male and female C58 mice and C57BL/6 mice housed with social and physical EE. EE condition added chewable toys, climbing platforms, tunnels, and running wheels. Females and males in non-enriched cages had a higher frequency of jumping behavior (more than 15,000 and 10,000 jumps per night, respectively) (77). Additionally, these authors reported that EE affects the microstructure in the gray matter, cerebellum, hippocampus, and striatum by enhancing synaptic plasticity and mitigating stress (78) (aspects that are out of the scope of the present review).

Providing EE also aims to improve the environment's utilization and the animals' coping abilities, reducing the endocrine and behavioral reactivity to challenges (6, 37, 75). A better ability to habituate to challenges was observed in Long Evans and Sprague-Dawley rats of 6 months old when exposed to 6 weeks of physical EE protocol (large cages with running wheels, climbing ropes, among other objects) (25). In this study, when facing the forced swim test, enriched animals had a higher frequency of swimming without struggling (131%) and grooming than control rats (71%) (25). Swimming without struggling is associated with enhanced coping abilities in rodents and decreased stress (79), which is a response elicited by EE.

The open field test (OFT) is another test used to measure exploratory behavior and activity in rodents after an EE protocol. A study evaluated male and female Long Evans rats at 34 PND after receiving physical EE (larger cages) for 30 days (80). It was found that enriched rats interacting with shoestrings, tennis balls, and hanging objects spent 56.6% of their time in the center and less time in the outer edge (18.7%). Additionally, EE animals entered 137% more central squares than control rats reared in plastic shoeboxes. In the OFT, thigmotaxis (the tendency to stay close to the walls of the arena) is an index of anxiety or fear, while staying in the center of the field indicates exploratory behavior (49, 81). Thus, the results of the previous study suggest an increased exploratory behavior by inhibition of the fear response to novel environments (80). This is similar to what other studies have found, where Fernández et al. (82) reported in male Sprague-Dawley rats at 20 months old. EE provided as larger cages, groups of 10 rats, and the addition of tunnels and toys for 60 days significantly increased exploration behavior in the OFT, considered behavioral flexibility.

In adult Sprague-Dawley rats (2-month-old), Pham et al. (83) compared enriched conditions (eight animals per cage, in wire mesh cages with tunnels, ladders, ceramic pots, wooden planks, among others) with control housing (isolated animals in standard plexiglass cages) provided for 12 months. Through the OFT, non-enriched animals were more active (activity counts above 120) and groomed less (6.6%) than enriched rats (~80 activity counts and 94%, respectively). Likewise, physical (plastics tubes) and feeding EE (sunflower seeds) provided to male Wistar rats of 200 g for 10 days increased the average time spent in the center of the OFT when compared to controls (51 vs. 13.33 s, respectively), and was lower than controls in the peripheral areas (239 vs. 285 s, respectively) (31).

The evaluation in the OFT in mice has shown similar results as reported by Manosevitz (84) in male and female mice of an inbred strain exposed to larger cages, hardware-cloth platforms, nesting material, and wood mazes (physical EE). When compared with control animals (reared in standard cages with nesting material), higher activity scores were recorded in enriched female and male mice (38.81 vs. 58.52, respectively). Similarly, the same researchers established that enriched mice (using the same EE and control conditions as the previous study) were 26% more active than control mice in the OFT and had lower defecation scores (up to 2.1 ± 1.2 vs. up to 2.7 ± 1.5) (85). Additionally, EE males and females also ran 40% more than control mice when evaluated using running wheel scores, which suggests that enriched mice have better adaptation skills to novel stimuli than animals raised in restricted environments. These responses are closely related to the effect that the rearing conditions have on the animals' development, as mentioned by Manosevitz and Pryor (86) in C57BL/6J strain mice. These authors compared small-wire, small-Plexiglas, large-sire, and large-Plexiglas cages and their effect on the animals' BW, OFT activity, defecation, and water consumption. All animals receive cotton balls as nesting material. The results showed that animals reared in larger cages were heavier, 16% more active in the OFT, and defecated 2.2 times less than those reared in small cages. Thus, physical EE involving cage size highly influences the response of mice.

Results in the elevated plus maze (EPM) agree with other behavioral assays since the EPM evaluates anxiety-like behaviors by measuring the number of entries to the open or closed arms (53, 87). In this sense, increased anxiety is indicated by a significant activity in closed arms, while an anti-anxiety behavior is related to an increase in entries to open arms (81, 88). Dandi et al. (49) found behavioral differences in adolescent (23 PND) male and female enriched and non-enriched Wistar rats (EE for 10 weeks) and subjected to acute and chronic stress protocols that included restrain, cage rotation and tilt, social isolation, and food and water deprivation, among others, as stressors. Enriched animals were housed in groups of five in large cages with tunnels, non-chewable toys, platforms, and running wheels. In contrast, non-enriched rats were housed in groups of two in standard laboratory cages. Regardless of the stress protocol, the percentage of open arms was higher in enriched animals (between 30 and 35%) than in the standard (15–27%). In contrast, the number of closed arm returns was significantly lower in the enriched group (between 1.5 and 2) than in animals housed in standard cages (up to 4 times). In the FST, EE reduced immobility time (between 80 and 110 vs. up to 170 in control groups), and in the OFT, enriched rats spent more time in the center (up to 20 s) vs. non-enriched animals (as low as 5 s) (49). Muthmainah et al. (53) also compared the effect of a control group of 42-day-old male Wistar rats (exposed to chronic stress, including predator noise, cage tilt, cold swimming, among other stressors) with those exposed to chronic stress but with social (group housing) and physical EE for 21 days (large cages with balls, slides, running wheels, plastic tubes, bedding material, and ladders). The authors found that rats exposed to the stress paradigm spend less time in open arms (~20 s) than enriched rats (60 s). In addition, enriched rats spend significantly more time in the open arms than the control animals (15 s). Similarly, the number of entries was higher in EE animals (~5 vs. up to 2, respectively). Together, these results indicate that EE increases the ability to habituate to novel environments and decreases the level of anxiety and fear.

**Figure 2:**
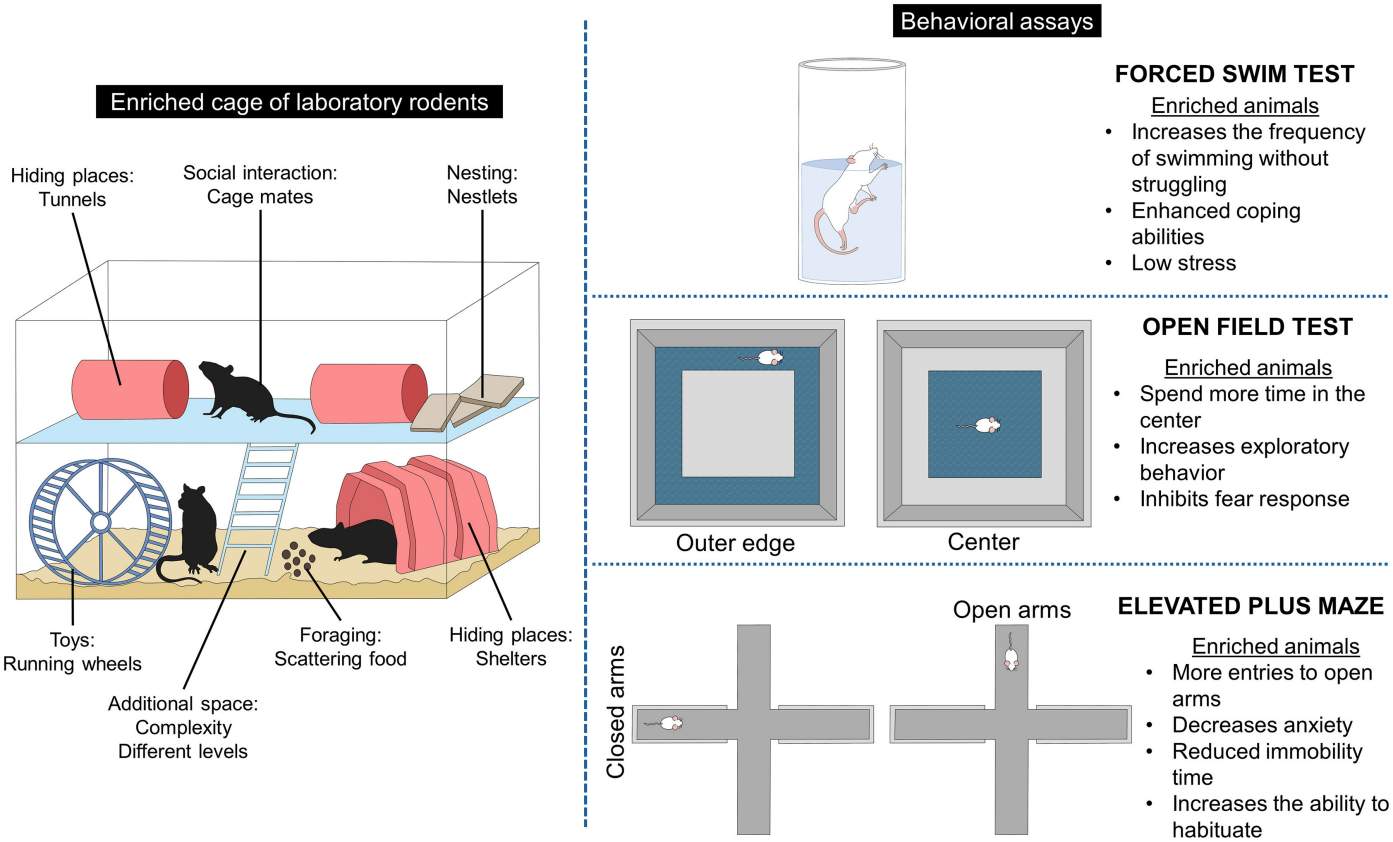
Effect of environmental enrichment on rodents' behavioral response.

Through the EPM, McQuaid et al. (32) reported that male CD-1 mice at 21 PND receiving social and physical EE for 6 weeks (large cage with two running wheels, shelters, tunnels, and cotton nestlets) had fewer entries in the closed arms, suggesting that EE reduces novelty fear. Similarly, a decrease in anxiety-like behaviors was reported in BALB/c mice reared under enriched or control conditions from birth to PND 56 (89). Enriched mice received running wheels and tunnels, while control animals remained in standard opaque plastic cages. In both strains, enriched animals entered more rapidly (latency time between 80 and 200 s) and spent more time in open arms (between 40 and 120 s). Mesa-Gresa et al. (26) compared a group of chronic social stress (house changing with unfamiliar mice) in NMRI male mice at 83 PND with an EE group receiving enrichment up to PND 112. In the EPM, animals enriched in groups of four in larger cages containing a running wheel and assorted toys spend less time in the closed arms than standard animals, an anti-anxiety behavior, and an indication of faster habituation to new environments. Moreover, in the novel object recognition test, mice housed under EE conditions had a higher frequency of grooming, a measure of body care. Additionally, Ramírez-Rodríguez et al. (90) found that EE (tunnels and running wheels) provided constant or gradually decreased anxiety-like behaviors in adult male Balb/C mice exposed to EE for 42 days. Figure 2 summarizes the main behavioral responses of laboratory rodents to EE.

The use of EE in laboratory rodents is intended to improve animal welfare. However, some studies have reported increased aggression when provided with EE, particularly in mice (55). For example, male CD-1 mice (21 PND) group-housed (groups of 3 mice per cage) and enriched with large cages with running wheels, shelters, tunnels, and cotton nestlets for 6 weeks had more wounds and excessive aggressiveness (32). McQuaid et al. (91) also reported that although CD-1 male mice enriched with running wheels, shelters, and tunnels for 2 and 4 weeks at 42 to 56 days old were more active (between 1,800 and 1,700 activity counts), the number of attacks, aggressive chasing, and aggressive grooming also increased when compared with control animals (those receiving only one cotton nestlet). Similar results were found by Haemisch et al. (92) in 120-day-old adult male DBA/2J mice housed in enriched conditions with physical (removable PVC platform with an underneath labyrinth) and social (three animals per cage) EE. By evaluating attacking behavior against intruders, the authors reported that EE animals showed a higher number of attacks (up to 25 attacks/30 min) than standard animals (maintained in cages with soft bedding, approximately four attacks/30 min). Moreover, aggression increased with time, reaching its peak after 6 weeks of EE. Increased aggression was also found in 70-day-old male CFLP mice reared in cages with physical enrichment (shelves and nestboxes), which was accompanied by higher testosterone levels (13.07 ± 1.89 ng/ml) than mice reared in unmodified cages (without shelves and nestboxes) (93). These studies show that enriching cages with objects that are defendable may exacerbate aggression in animals, which might reflect a competitive state to access EE resources. However, further studies are required to elucidate the relationship between EE and increased aggression, as other studies have found significant reductions in aggressive interactions when 7-week-old male BALB/c mice were provided with tubes, nestboxes, wooden blocks, and tissue paper bedding as physical enrichment (94). Moreover, other authors such as Armstrong et al. (95) highlight that physical EE (corn-husk nesting material) decreased aggressive (less visible wounds) behavior in BALB/cAnNHsd mice during the initial introduction of the enrichment (four days), but resulted in no significant differences by day seven when compared to standard animals (bedding only).

## 6 Additional considerations of environmental enrichment

As discussed, EE protocols have several positive effects on laboratory rodents. However, authors mention that these effects depend on several factors such as enrichment-related (i.e., type of EE, duration of EE) and animal-related aspects (i.e., strain, sex, or age) (25, 29).

In the first instance, the definitions of enrichment and “standard or control conditions” are inconsistently used in the literature. As shown in the present review, the characteristics of control conditions or un-enriched cages greatly differ among studies. While some authors refer to EE as the provision of nesting material, other studies consider this as standard housing, which difficult the standardization of terms (6, 74). Moreover, not every study provides a detailed description of control conditions. Some studies only state that control animals were housed in “standard laboratory cages”, without specifying if they include bedding materials or refer to completely barren cages. Similarly, when considering social enrichment, some studies refer to “group-housing” without specifying the number of animals per cage, which makes it difficult to replicate the methods in other research facilities. Additionally, as Veissier et al. (7) mention, it is essential to differentiate between environmental improvement from EE. The first provides immediate satisfaction of behavioral needs, while the effect of EE is observed in the long term, with animals acquiring the skills to better adapt to their environment.

Most studies address the issue of “standardizing” the use of EE protocols inside research facilities. However, there is also an inconsistency in reporting the type of EE and the provided items (e.g., nesting material, shelters, cage mates, food/treats, tunnels) (19). For example, it is still unclear how often the items must be changed. Some studies recommend changing them once per week to avoid stress associated with novel objects, while others recommend two or three times per week to avoid habituation to the items (91). Moreover, when applying EE protocols, instead of a single element, the interaction between several enrichments seems to have a bigger effect on animals (52). The reviewed studies in the present manuscript suggest that physical and social enrichment are the main types of enrichment used for laboratory rodents. In particular, according to the data consulted, positive behavioral and physiological responses are mostly observed when rodents receive nesting materials, hiding places, additional space, and when they are housed in groups. The enhanced response of animals to these types of enrichment is due to their importance in promoting species-specific behaviors, meeting their biological needs, and the nature of rodents as a social species.

Regarding the strain and animal-related factors, some studies have found that Sprague-Dawley rats were more active than Long-Evans rats even when provided with the same EE (25). Additionally, animals have preferences and tend to use some items more than others. According to Ratuski and Weary (6) metareview, for rats, social housing is highly recommended (17 articles), followed by larger/higher cages (10 articles), nesting material (nine articles), shelters/nest boxes (11 articles), and foraging opportunities (seven articles). In the case of mice, providing nesting material was the main EE (19 apers), followed by social housing (14 articles), shelters/nest boxes (12 articles), foraging opportunities (seven articles and an average active use of 52.6%), and larger cages (six articles) (6, 13). This implies that animal strain and preference must be considered when adopting EE in laboratory rodents. This was addressed in Van de Weerd et al. (96) study, where six different nesting materials were evaluated in 56 to 70-day-old male and female C57BL/6J and BALB/c mice. When comparing paper towels, Kleenex^®^ tissues, Enviro-dri^®^, cotton strings, wood-wool, and wood shavings, mice preferred cages with tissues and towels over wood-derived materials. Additionally, Hobbs et al. (97) found that single-caged mice enriched with marbles, tunnels, and nestlets spend more time interacting with the nesting material (271 min). Likewise, a mean duration of 35 min digging the bedding suggests that both elements are preferred enrichment items. Thus, as previously mentioned, although the addition of nesting material is recommended (according to the published data), the type of material must be selected according to the animals' preferences.

Sex is another aspect that highly influences the response of animals to EE. Kentner et al. (19) found that, from 681 papers revised, only 100 used both sexes to assess the effect of EE, while more than 450 papers were focused on males. Similar results were obtained by Cait et al. (2), who found that 59% of studies addressing EE focused on males only, 35% on females, and only 4% on both sexes. The justification for not including females is due to the hormonal reproductive cycle fluctuations that affect behavior (37). However, authors have stated that this perspective is incorrect and comparisons between males and females are needed to “standardize” EE protocols (19).

Finally, one of the main issues with EE provided to laboratory rodents is the alterations or variations that EE might pose to research outcomes. Several researchers address that EE might compromise the integrity of the research, leading to data variation and poor reproducibility of the results (19, 52). However, even under standard housing, every research facility has different concepts of “standard” housing, husbandry, and management conditions. Thus, variations in these elements would result in inaccurate results regardless of the addition of EE (9). Likewise, as shown in the present review, rodents reared in non-enriched conditions exhibit endocrine, physiological, and behavioral deficiencies that can negatively affect the experimental protocol (9). For example, studies have found that control animals have higher coefficients of variation than enriched rodents when assessing overall behavior (control CV: 0.67 ± 0.06 vs. EE CV: 0.56 ± 0.04) (25). Therefore, as Schrijver et al. (52) mention, adding EE to research protocols can increase the results' sensitivity and reproducibility, especially when studies provide a detailed protocol so other authors can replicate the experimental conditions.

## 7 Conclusions

Improving the housing conditions for laboratory rodents has gained relevance to meet the animals' biological needs and preserve their welfare. However, in some instances, housing conditions in research facilities marginally fulfill these needs and limit the presentation of natural behaviors. When rodents cannot express highly motivated behaviors such as nesting or burrowing, negative mental states such as boredom or stress might arise. EE is a collection of physical, sensory, cognitive, and/or social stimulation greater than the one received under standard housing conditions aimed to prevent said states. Providing EE to laboratory rodents has shown several endocrine, physiological, and behavioral benefits to animals. In the first instance, CORT concentrations (a stress biomarker) decrease in rats and mice exposed even to short periods of EE. Other physiological parameters, such as tachycardia, hypertension, and shorter HRV, are ameliorated with the implementation of EE (reflecting a beneficial effect). Finally, rodents reared under EE conditions have a higher presentation of anti-anxiety behaviors, together with a reduced fear response and increased exploratory behavior. These results show that EE provides animals with more substrates to meet their behavioral needs and, thus, reduces the potential stress that confinement might represent. Although there is a lack of EE standardization across protocols, it is essential to consider providing enrichment for laboratory rodents, as recent studies have shown that it does not affect the integrity or reproducibility of the results. Nonetheless, factors related to the type of EE and the animals must be considered to establish an EE program aiming to improve the welfare of laboratory rodents.
